# Copper Cement Chemistry

**Published:** 1915-04

**Authors:** W. S. Medell


### COPPER CEMENT CHEMISTRY.

BY W. S. MEDELL, B.SC.

*Read before the Lehigh Valley Dental Society, at its fall meeting, Mauch Chunk, Pa., September* 21, 1914, *and published in the Items of Interest for March,* 1915.

"The vagueness and mystery surrounding the products which the dentist uses often enhanced by most confusing advertising gymnastics, are not a tempting barrier, with ever so fruitful a field behind it. It is a mistake, in my opinion, from both the scientific and commercial standpoint, to withhold enlightenment, and it is equally difficult to conceive any but good results by closer communion between dentist and manufacturer."

This paper is opened with the above paragraph, quoted from a paper on the chemistry of oxyphosphates read before the Colorado State Dental Society in June, 1902, and published in *Items of Interest.* It fits the present situation of the copper cement question like a glove. The object of this paper is to lay the cards on the table face up, so as to give the dental profession an opportunity to judge the situation in the light of full knowledge.

You have no doubt read many advertisements on copper cement in which this statement or its equivalent appears: "In order to clear the situation we give the following information." Upon reading the advertisement you have obtained about as much real satisfaction as did the old negro who was sleeping beside the fire while the 'possum was cooking. His young grandson came in and proceeded to eat the meal the old man was dreaming about. After disposing of everything in sight the youngster placed the bones and remnants on the table by his grandfather's side, rubbed grease on his mouth and fingers and departed. Presently the old man awakened, and, remembering the joys in store, proceeded to business. He at once discovered the state of affairs. He looked at the bones, examined his fingers, licked his lips and exclaimed, " 'Fore de Lord, I done forgot I ate him."

### AMES'S COPPER CEMENT.

Dr. Ames was the first to introduce copper cement, and all other black copper cements are imitations of his product. I am sure there is not one exact reproduction on the market. Copper cements containing about twenty-five per cent. black oxide of copper were introduced a number of years ago in imitation of Ames. They were and are inferior to the original in many respects. The copper oxide content is not properly made.

### RED COPPER CEMENT.

About ten years ago Dr. Fleck introduced red copper cement, this being the first one other than black. Its use finally resulted, less than a year ago, in an imitation being placed on the market by a prominent cement manufacturer, who at the same time introduced what is called "white copper" cement. Since then another manufacturer brought out so-called copper cements in all the colors of the rainbow. So much for the history of introduction of this class of oxyphosphates and their imitations.

### DENTAL CEMENTS.

Dental cement and oxyphosphate cement are synonymous terms, because there is not a dental cement of any value whatever which is not an oxyphosphate. Crown and bridge, inlay, temporary filling, silicate and copper cements are all oxyphosphates. They are all of the same general form, which is an oxide powder and a phosphoric acid liquid. Therefore oxyphosphate of copper must have its copper content in the form of oxide, otherwise it is not a copper cement. That is one in which the copper present has true cementing action. Such a cement can be only one of two colors, black or red, because Nature provides but two oxides of copper, cupric oxide, which is black, and cuprous oxide, which is red. To call any cement a copper cement simply because it has a small percentage of some copper salt in the powder or the liquid is a misrepresentation. The manufacturer of such an article is sailing under false colors. If his product had "all the characteristics of the black copper, except appearance," and was "the result of years of investigation of the copper cement question by real scientists," he would be more than glad to call it by its right name and distinguish it from all inferior grades. He would surely not state in the same advertisement which extolled its virtues that the black would continue to be used "where the color is not objectionable and a pronounced preservative effect is desired," and with the next breath add: "The formula (of white copper cement) calls for a minimum of copper to give the full sedative and bacteriological effect."

### RED COPPER CEMENT.

It is true that red is less objectionable than black as to color, but the main advantage is that it does not discolor tooth structure. At the same time it has all the cementing antiseptic, sedative and preservative value of the black, because it is of the same type of construction. Therefore there is no object in producing a red copper cement unless cuprous oxide is used wholly or in part. The imitation, mentioned above, is simply zinc oxide cement containing a small percentage of some copper salt, and does not owe its color to the presence of cuprous oxide, but to some other coloring material; probably iron oxide, which as no value except its coloring power. Red oxide of copper when burned in the air is changed to black oxide. All that is necessary to prove the truth of the above is to burn some of this cement. It will not turn black. The original red copper cement powder does turn black on being burned.

Any chemist will bear witness to the truth of the above statement.

The manufacturer introducing the Joseph's coat color scheme is the only one who makes any attempt to prove the germicidal properties of his product. He publishes a letter from the man who did the work, which describes the method used in testing, and says: ''Three other brands of copper cement were tested in the same manner.'' Just why he makes this statement is hard to discover, because he fails to give any results by which to make comparision. He probably omitted these results for very good reasons. The letter further states: ''During this work another advantageous property of your cement was noted, in that there was almost no diffusion into the surrounding media of the soluble copper salts, which might be both poisonous and irritating.''

This statement most surely discloses the writer's lack of knowledge in regard to the subject. It is a well-known fact, demonstrated by years of dental practice, that true copper cements, even when containing over ninety per cent. of copper oxide in the powder, are decidedly non-poisonous and far from being irritating, nay, are markedly sedative in their action. Furthermore, the above tests were completed in two days. What guarantee can they give that the cement will retain its germicidal properties indefinitely?

A dentist, who is a personal friend of the manufacturer, reports as follows: ''For more than a year I have been testing this material in every conceivable way . . . and the only point I felt was not yet settled by these tests was that of its germicidal property, and my report to you has been delayed on this account. I have felt that sufficient time had not elapsed to determine this point from mouth tests. Now, however, since the report sent me (the one referred to above) is so conclusive as to definite germicidal action, I feel that this point should be considered settled.'' His personal friendship is quite evident when he is willing to substitute a two-day test for lack of results in his own experience of "more than a year, . . . testing . . . . in every conceivable way."

### BLACK COPPER CEMENT.

The manufacturer of the original black copper cement has carried on a campaign of advertising claiming that a copper cement which is a combination of zinc and copper will have no value imparted by its copper content, even though it be an oxide, unless there is present practically one hundred per cent. of such oxide. The advertisements put out in this campaign were confined to *claims* up to a month or two ago, when reference was made to an article by Dr. W. V-B. Ames in the following words: "We invite the checking up of the work of Dr. Ames, published in the *Dental Review,* June, 1914, by any 'Institute of Industrial Research,' or any scientific individual." After reading the article in question I decided to accept the invitation on the grounds that I am a "scientific individual," whatever that means. Not being a bacteriologist, I shall confine myself to a discussion of the chemical side of the paper. On pages four and five of the reprint of the article the following statements appear:

"Most important of all, from the standpoint of proper understanding of what may be expected from admixture of cement-making oxides, is the information gained by . . . . analyzing the soluble salt produced and available for analysis in the fresh cement.

"Chemically . . . ., it happens that the salt formed during the mix is at the expense of the zinc oxide, which fact forthwith removes all chance of discussion. Copper oxide, properly added to a zinc oxide cement, will give added integrity of mass . . . .

"The blending of zinc and copper oxides and then subjecting the mixture to the action of an acid phosphate solution is analogous to placing sheets of metallic copper and zinc in an acid solution to constitute the galvanic battery. In such a battery the solution of metal is entirely at the expense of the zinc, just as in this cement all salt formed is at the expense of the zinc oxide, except when nearly 100 per cent. oxide is contained.

"With mixed oxides the copper salts formed after setting will be less in proportion to zinc salts than the relative proportion of copper oxide to zinc oxide in the mixture, or entirely absent. . . .

"It is easy to demonstrate that there is nothing else than the zinc salt formed in such a zinc copper mixture unless the copper content reaches so close to 100 per cent. that there is no argument in favor of the zinc oxide admixture. Chemical analysis of solutions obtained by puddling freshly mixed cement in water, easily shows evidence that metallic oxides obey the same law which governs the corresponding metals in entering into combinations. Metallic oxides, like the corresponding metals, obey the rule of electro-chemical potentiality in entering into combinations. The oxides interesting to us as cement ingredients occupy relations as follows, starting with the most positive, *i. e.*, zinc, iron, cobalt, copper, mercury, the zinc being positive to iron, the iron to cobalt, the cobalt to copper, and copper to mercury, each metal or its oxide being dissolved when subjected to acid in the presence of one to which it is positive.

"Chemical analysis will show that there is no copper salt formed when either cobalt, iron or zinc oxides are mixed with copper oxide, unless the copper predominates to the extent of nearly 100 per cent."

### TESTS BY THE AUTHOR.

Demonstration is better than explanation. Therefore, I will perform a few experiments in checking the above statement. First, consider analyzing the soluble salt formed in the fresh cement and the claim that in a mixture of zinc and copper cement-making oxides the salt formed during the mix is at the expense of the zinc, and that no copper salt is produced unless nearly 100 per cent. of copper oxide is present. Take the five cements before us. No. 1 is so-called "white copper," No. 2 is the light shade out of the rainbow, No. 3 is black copper made in imitation of Ames and is supposed to contain 25 per cent black oxide and 75 per cent. zinc cement; No. 4 contains 25 per cent. black oxide and 75 per cent. zinc cement; and No. 5 is red copper containing 25 per cent. red oxide and 75 per cent. zinc cement. A mix of each one is made and puddled with water and allowed to stand for a time, as you see, then filtered and ammonium hydrate added in excess to the filtrate. We have no characteristic blue shade in No. 1 or No. 2; all the others, which are of the mixed copper-zinc type, do show the blue. This is contrary to the article quoted. It is here demonstrated that soluble copper salt *is* formed even where the copper oxide content is much less than the zinc, and none where copper salts are used in the place of oxides.

It is also stated that the addition of copper oxide to a zinc cement improves its structure; also that the copper salts formed after setting are less in proportion to zinc salts than the original proportion of copper to zinc. This, taken in connection with the other statements, clearly indicates that the author of the paper reasons that no acid phosphates of copper are formed under the conditions mentioned, but that oxyphosphate of copper is finally formed. It can be demonstrated to the satisfaction of any chemist that the reaction involved between powder and liquid in all oxyphosphate cements proceeds in the following manner:

### MIXING CEMENTS.

The first additions of powder form acid phosphates, which are soluble; as more and more powder is added saturated phosphates are formed, the same being insoluble; and finally, when the last quantities of powder are added, excess oxide is present and we have the oxyphosphate.

Then if no soluble acid phosphates are formed at first there will be no oxyphosphates formed after setting has taken place, because the nearly neutralized acid present, after setting, will not react under conditions which prevented the action of the unneutralized acid in the first place.

Again: "Chemical analysis shows that metallic oxides obey the same law which governs the corresponding metals in entering into combinations." Take some metallic copper, add to it some dilute phosphoric acid and boil. What happens? As far as chemical reaction is concerned, nothing. The metal does not go into solution. On the other hand, take some copper oxide, treat it in the same way with phosphoric acid. Solution takes place. Not only does solution take place, but it takes place very quickly. Take some red oxide of copper, treat it like the black. Now we see a change; the color is darker and on filtering we find there is copper in solution. What has happened? Simply this: In cuprous oxide there are two atoms of copper; when treated with phosphoric acid these two atoms react on each other; one is oxidized to the cupric state, the other is reduced to the metallic state. The first goes into solution as cupric acid phosphate, the second remains behind as insoluble metallic copper, and we have the proof, in one and the same reaction, that oxides do not enter into combination in the same manner as the corresponding metals.

In regard to one metal or its oxide being dissolved in acid in the presence of one to which it is positive, let us see. To a mixture of zinc and copper filings, 75 per cent. of the first and 25 per cent. of the second; add some dilute nitric acid, then an excess of ammonium hydrate. Copper is in the solution. To an alloy of zinc, between 85 per cent. to 90 per cent., and the balance copper, add some dilute nitric acid, then ammonium hydrate. Copper again in solution. In the list of metals which interest us as cement ingredients, aluminum is not mentioned. Why? For the following very good reasons: Into an acidulated solution of copper drop some metallic aluminum, boil and observe that all the copper is deposited. Pour off the liquid, wash with water, add dilute nitric acid and observe that the copper goes into solution, the aluminum does not.

Lastly, he states that in mixtures of iron and copper oxides there is no copper salt formed unless the copper predominates to the extent of nearly 100 per cent. Take a mixture of iron oxide 75 per cent. and copper oxide 25 per cent. Boil with nitric acid, filter and add ammonium hydrate. Large quantities of copper prove to be present and very little, if any, iron.*

* All chemical experiments mentioned in the above paper were performed by its author before the Lehigh Valley Dental Society on the day it was read, thus demonstrating the scientific exactness of the statements made.—J. C. Hertz, Chairman of Committee.

